# Intraepidermal Nerve Fiber Density as Measured by Skin Punch Biopsy as a Marker for Small Fiber Neuropathy: Application in Patients with Fibromyalgia

**DOI:** 10.3390/diagnostics11030536

**Published:** 2021-03-17

**Authors:** Mary A. Kelley, Kevin V. Hackshaw

**Affiliations:** 1 Department of Neurology, Dell Medical School, University of Texas at Austin, 1601 Trinity St, Austin, TX 78712, USA; 2 Department of Internal Medicine, Division of Rheumatology, Dell Medical School, University of Texas at Austin, 1601 Trinity St, Austin, TX 78712, USA; Kevin.Hackshaw@austin.utexas.edu

**Keywords:** fibromyalgia, small fiber neuropathy, epidermal nerve fiber, biomarker, central sensitization

## Abstract

Small fiber neuropathy (SFN) is a type of peripheral neuropathy that occurs from damage to the small A-delta and C nerve fibers that results in the clinical condition known as SFN. This pathology may be the result of metabolic, toxic, immune-mediated, and/or genetic factors. Small fiber symptoms can be variable and inconsistent and therefore require an objective biomarker confirmation. Small fiber dysfunction is not typically captured by diagnostic tests for large-fiber neuropathy (nerve conduction and electromyographic study). Therefore, skin biopsies stained with PGP 9.5 are the universally recommended objective test for SFN, with quantitative sensory tests, autonomic function testing, and corneal confocal imaging as secondary or adjunctive choices. Fibromyalgia (FM) is a heterogenous syndrome that has many symptoms that overlap with those found in SFN. A growing body of research has shown approximately 40–60% of patients carrying a diagnosis of FM have evidence of SFN on skin punch biopsy. There is currently no clearly defined phenotype in FM at this time to suggest whom may or may not have SFN, though research suggests it may correlate with severe cases. The skin punch biopsy provides an objective tool for use in quantifying small fiber pathology in FM. Skin punch biopsy may also be repeated for surveillance of the disease as well as measuring response to treatments. Evaluation of SFN in FM allows for better classification of FM and guidance for patient care as well as validation for their symptoms, leading to better use of resources and outcomes.

## 1. Introduction

### Small Fiber Neuropathy

Small fiber neuropathy (SFN) is defined as a type of neuropathy that occurs from damage to the small peripheral nerve fibers. SFN predominantly or exclusively affects the unmyelinated C-fibers, sympathetic and parasympathetic autonomic axons and the thinly myelinated A-δ sensory axons. Impairment of these nerve fibers results in the clinical condition known as a small-fiber neuropathy (SFN). This pathology may be the result of metabolic, toxic, immune-mediated, and/or genetic factors. SFN may be the primary disease, or it may be a secondary process from an underlying disease state. It can present as a diffuse pain syndrome and be non-length dependent unlike typical neuropathies. Historically, somatic and autonomic neuropathies were classified separately, however, it has come to be known that C-fibers also have important effector and trophic functions, e.g., controlling sweating, bone growth, immunocytes, and the microcirculation. Therefore, the more inclusive “small fiber” terminology became preferred [1]. 

Different types of small-fibers express different arrays of neuropeptides and innervate many different types of cells, tissues, and organs, therefore SFN typically presents with varied symptoms. Most commonly neuropathic pain and sensory abnormalities are seen in a length dependent distribution, though this is not always the case. Patients will complain of constant or intermittent pain (burning, stabbing, electrical shocks or shooting, tingling, hyperesthesia, allodynia, or dysesthesias) [2] or they may not have any pain at all. They may have autonomic complaints exclusively, or an overlap of somatic and autonomic symptoms. The symptoms are at times unacknowledged due to their non-specific nature such as fatigue, daily performance decline, anxiety, and depression [3]. Additional common complaints are new difficulty performing usual activities (exertional intolerance and chronic fatigue), postural orthostatic hypotension and tachycardia (POTS), postprandial bloating or nausea, diarrhea and/or constipation (irritable bowel), urological complaints, and chronic daily headache. Pathological studies have also shown small-fiber dysfunction to result in malfunction of the body’s small blood vessels, which results in muscle hypoperfusion, deep aching pain, and exercise intolerance [4,5].

Clinical evaluation remains at the forefront of diagnosis, however, given the variability and non-specificity of small-fiber symptoms, the case definition includes a requirement for objective “biomarker” confirmation. Small fiber dysfunction is not typically captured by diagnostic tests for large-fiber neuropathy (nerve conduction and electromyographic study). Therefore, skin biopsies are the universally recommended objective test for SFN, with quantitative autonomic function testing, and corneal confocal imaging as secondary choices. The forementioned techniques have for the most part replaced sensory nerve biopsy as it cannot differentiate afferent and efferent autonomic from somatic axons and is much more invasive [1,6,7,8,9].

The small nerve fibers have the highest regenerative capacity and prompt diagnosis and initiation of disease-modifying treatment of SFN can result in substantial regeneration and recovery, particularly in the young and otherwise healthy [10,11]. Evidence-based recommendations have been developed for blood-test screening of patients with SFN as well as genetic testing with a list available at https://neuropathycommons.org/ (accessed on 12 March 2021). Typical causes include diabetes mellitus, autoimmune (e.g., celiac disease, sarcoidosis, Sjögren’s syndrome), infectious (e.g., human immunodeficiency virus, hepatitis C), alcohol dependence and exposure to toxins, nutritional deficiencies (e.g., vitamin B12 deficiency), amyloidosis and paraneoplastic syndromes, and hereditary causes (e.g., Fabry’s disease, sodium channelopathies) [2]. Table 1 provides a more comprehensive list of causes. However, comprehensive testing fails to identify a cause in 20% to 50% of patients with SFN [1,3,9,12]. As we study the various diseases in which SFN is observed we are better able to understand the mechanisms that may be implicated in these idiopathic/cryptogenic cases of SFN.

## 2. Methods

The following is a narrative review that encompassed use of the search engines PubMed and Google Scholar. Search terms used included Fibromyalgia, Fibrositis, Widespread Pain, central sensitivity, small fiber neuropathy, epidermal nerve fiber, epidermal nerve fiber density. Period scanned was from 2001 to December 2020.

## 3. Evaluation of SFN

### 3.1. Skin Biopsy

Skin biopsy is a minimally invasive technique used to diagnose SFN, with a sensitivity up to 94% and a specificity up to 97% [6,7,13,14,15,16,17]. Though nerve conduction studies and electromyography is beyond the resolution of small fiber neuropathy, it may be beneficial in ruling out large nerve fiber damage which can be present in SFN. Currently skin punch biopsies are the preferred method for objective evaluation of SFN. The procedure was initially developed at the Karolinska Institute and was later standardized at the University of Minnesota and Johns Hopkins University and allowed for a greater ability to diagnose and study SFN. The technique utilizes antibodies to the protein gene product 9.5 (PGP 9.5), a neuronal form of the ubiquitin carboxyl terminal hydroxylase transported by the slow component of axonal transport. The European Federation of Neurological Communities recommended a skin biopsy with staining of PGP 9.5 as a grade A recommendation [13,18]. Diagnosis of SFN is made when nerve fiber density is in the lowest 5th percentile. Normative values for Intraepidermal nerve fiber density (IENFD) have been determined for both age and gender as well as children. Guidelines for the use of skin biopsy in clinical practice have been published as well, providing standardization of the technical procedures, including IENFD count, internationally [7,13,14,15,19,20].

Skin biopsies can be performed in any medical setting and mailed in Zamboni fixative, to an accredited neuropathology lab for quantification of IENFD and interpretation based on well-established normative data. The tissue samples are vertically sectioned and immunolabeled against PGP9.5, the best pan-axonal marker. PGP9.5 darkens and magnifies the small-fibers which allows the IENFD to be measured with standardized methods [18,21].

The procedure is simple and utilizes common tools found in a clinical setting. Side effects are minimal with minor inflammation and bleeding being the most common though are easily remedied. Skin punch biopsies can be performed anywhere on the body though the most common sites are typically on the distal leg at 10 cm above the lateral malleolus, the proximal leg 10 cm above the lateral epicondyle of the femur, 20 cm distal to the iliac crest and the dorsum of the foot. These sites in particular have the most data available on them and therefore have well established age and gender adjusted normative values. Table 2 provides a step by step description of a typical biopsy.

Advantages of the biopsy include reproducibility and the ability to repeat as needed, and, unlike nerve biopsy, it does not result in sensory disturbances or long-term pain at the site of biopsy. The perceived disadvantages of biopsies include cost, lack of equipment at clinics, and lack of familiarity with the procedure by providers. However, many of these perceived disadvantages may not be relevant as providers become more familiar with the process, something that this article in particular hopes to convey. As this procedure has come to be accepted as the gold standard for the diagnosis of SFN it is now easily billable to insurance companies and labs are readily available to process the skin samples. Many labs will send the full biopsy kit with fixative and detailed instructions, requiring the performing clinic to only have to provide the local anesthetic.

#### Bright Field vs. Immunofluorescence

Samples are evaluated using either bright-field immunohistochemistry (BFI) or immunofluorescence (IF), the latter being the slightly more sensitive method. Visualization of the samples allows for evaluation of somatosensory and autonomic nerve fiber conditions. The length of the section is measured and then the number of the nerve fibers is counted, and the density of the nerve fibers in 1 mm is calculated, and comparison with age and gender-matched normative values is performed. The innervation of the sweat glands and quantification of axonal swellings may also be evaluated and offer insight into the pathophysiology of the disease.

Studies have demonstrated excellent correlation between measurements using the two methods, with a BFI/IF ratio of about 1:2. It was determined that both methods despite being on a different scale are able to accurately diagnose SFN. One study found that combining the two techniques (one or the other positive for SFN) did not increase the diagnostic performance. Inter-rater variation (0.4 ± 1.5 IENF/mm) has been observed in both and therefore values of IENFD very close to the cut-off of normal vs. abnormal are to be considered with caution before providing a diagnostic judgment and may require subsequent biopsies to confirm for or against a diagnosis [7,16,18]. Overall it has been observed that IF and BFI are comparable in assessing the morphometry of epidermal innervation and diagnosing SFN when referred to appropriate normative reference values.

Normative data for immunofluorescence: Age, gender and biopsy site showed an independent linear correlation with IENF density. For each decade the 5° quantile IENF cutoff showed a 0.54 fibers/mm decrease, while females exhibited a 1.0 fiber/mm cutoff greater than males. Compared to the lateral distal lower leg, biopsies from the calf showed a 3.4 fibers/mm lower 5° percentile cutoff, documenting a variation linked by site [14]. Normative datasets have been published and continue to be refined over time.

### 3.2. Autonomic Function Testing

Autonomic function testing (AFT) can be beneficial when patients are presenting with autonomic symptoms and therefore may be used in addition to a skin punch biopsy to further quantify the disease. Autonomic testing is a type of electrodiagnostic medicine and clinical neurophysiology testing that can be used to assess sympathetic and parasympathetic neural pathways. Autonomic testing is designed to evaluate various clinical scenarios that may involve small-fiber autonomic dysfunction. Testing often requires the use of a tilt table and other tools that are frequently only found at tertiary medical centers. It is the position of the American Association of Neuromuscular & Electrodiagnostic Medicine (AANEM) that, in order to perform and interpret diagnostic studies of the autonomic nervous system appropriately and ensure quality patient care, the individual performing the studies must be a physician with special training in diagnosis and treatment of disorders of the autonomic nervous system and in application of particular neurophysiologic techniques to study these disorders. These requirements often limit the availability of AFT for routine use.

Techniques used to study autonomic function are separated into three categories evaluating: cardiovagal for parasympathetic dysfunction, vasomotor adrenergic for sympathetic dysfunction in patients with syncope, orthostatic hypotension, postural orthostatic tachycardia syndrome, and postural dizziness, and sudomotor function which controls sweat. Multiple tests are often conducted in tandem depending on the specific complaints of the patient.

Tests for the evaluation of cardiovagal function include the heart rate response to deep breathing; Valsalva ratio; and postural change.

Tests for evaluation of sympathetic adrenergic function include continuous beat-to-beat heart rate and blood pressure response to a Valsalva maneuver; tilt table test; or active standing.

Tests for the evaluation of sudomotor function included quantitative sudomotor axon reflex testing (QSART); thermoregulatory sweat testing; induced silastic skin imprints; and sympathetic skin response. Sudomotor function may also be tested with devices such as Neuropad and Sudoscan.

### 3.3. Quantitative Sensory Tests

Quantitative sensory testing (QST) was the first tool used to diagnose SFN, it is based on the psychophysical examination of sensory nerve fiber functions through assessment of thresholds to various stimuli, including pressure, vibration, cold, warmth, heat, cold pain and heat pain. This approach enables identification of loss of function as well as gain of function phenomena, such as allodynia and hyperalgesia. Though it is widely used, it is rarely used alone given its variable sensitivity and specificity depending on methods used. It is particularly beneficial in obtaining the full somatosensory phenotype of a patient [22].

QST can be performed with a comprehensive approach of threshold determination and a multimodal approach including thermal and mechanical stimuli in order to improve their sensitivity. In a recent study warm and cold detection threshold (WDT, CDT) were assayed combining limits and levels methods at the dorsal foot bilaterally and at the dorsal aspect of the non-dominant hand. Then, the limits method (LIM) was used alone for WDT and CDT at proximal thigh, and for cold and heat pain threshold determination at all the sites. Abnormal sensations including errata sensation, thermal allodynia or hyperalgesia, and aftersensation were recorded for all the tests. Results were compared with published reference normative values and for direct comparison with a cohort of 99 age- and gender-matched healthy subjects who underwent the same QST protocol. Mechanical detection threshold was measured with a standardized set of modified von Frey hairs using the method of limits in five determinations. The test was conducted at all the sites where thermal stimuli were tested. QST achieved the highest performance when both warm and cold thresholds were performed at the feet using both the limit and level test which yielded 85.1% sensitivity and 80.9 specificity for SFN in the subjects [6]. Though it has the benefit of being less invasive, QST has been criticized for being time consuming and subjective given that it relies on patient’s individual reporting. Additionally, QST requires alert and cooperative patients and can be obscured by malingering or psychogenic conditions. More than 15 different methodological approaches have been developed limiting its standardization. For all of these reasons QST is considered as an additional diagnostic test that is best used with a well characterized clinical assessment and skin biopsy [19,23,24]. Though research continues on this diagnostic tool given that with training it can be cheap and easy to use at bedside [25].

### 3.4. Corneal Confocal Microscopy

Confocal corneal microscopy is a noninvasive technique for detecting small-fiber loss, and findings with this technique have been found to correlate with those of skin biopsy in small-fiber neuropathies [19]. The cornea is innervated by small nerve fibers of trigeminal origin that enter through the middle third of the stroma. These fibers are then visualized and quantified by using in vivo corneal confocal microscopy. It is a relatively new diagnostic tool that was developed as a research technique and has since become an emerging clinical resource. It provides various morphometric parameters to quantify corneal nociceptors and their changes over time. Use of this tool is limited in that it requires specific equipment and a trained technician that are often unavailable, thus it is primarily used in research at this time [6,26,27,28].

The technique has primarily been used to assess patients with diabetes mellitus, it has also been used to demonstrate small-fiber involvement in several other conditions, including CMT type 1A, hereditary sensory and autonomic neuropathy, Fabry disease, autoimmune neuropathy and chemotherapy-induced neuropathy. With the recent development of automated corneal nerve image analysis and, the establishment of reference values, corneal confocal microscopy is a reliable diagnostic tool for evaluating SFN. The exact role of the technique in the diagnosis of individual patients with suspected SFN in clinical practice is still yet to be determined and requires additional research [19]. Skin punch biopsy to evaluate IENFD along with QST and QSART remain the most frequently used methods for evaluation of SFN, Table 3 provides a summary and comparison of these methods.

## 4. Fibromyalgia

Fibromyalgia (FM) is a well-recognized and common disorder of chronic widespread pain. It is characterized by specific criteria set out by the American College of Rheumatology (ACR). Recognition of the condition has evolved over the years, the currently accepted criteria for FM are the revised Diagnostic Criteria for FM 2016. The 2016 revision states that a diagnosis of FM is made when the following three criteria have been met: (1) Widespread pain index (WPI) ≥ 7 and symptom severity scale (SSS) score ≥ 5 OR WPI of 4–6 and SSS score ≥ 9. (2) Generalized pain, defined as pain in at least 4 of 5 regions, must be present. Jaw, chest, and abdominal pain are not included in generalized pain definition. (3) Symptoms have been generally present for at least 3 months. The WPI and SSS scoring is outlined in Table 4. It is important to note that per the 2016 revision the diagnosis of fibromyalgia is valid irrespective of other diagnoses. A diagnosis of fibromyalgia does not exclude the presence of other clinically important illnesses [29]. The evolution of the diagnostic criteria is summarized in Table 5.

The term myalgia suggests the disorder derives at least in part from muscle. However, the co-existence of neuropathic features of pain, autonomic dysfunction and a burgeoning literature implicating small fiber axonal loss in the setting of FM, has raised the question of whether the pain in FM is at least in part a neuropathic phenomenon [1,21,30,31,32,33].

Research demonstrates biochemical and neurobiological elements of fibromyalgia which include: neurotransmitters, hypothalamic pituitary adrenal axis (HPA axis), inflammatory cytokines, monoaminergic pathways, opioid peptides, sex hormones, nerve growth factor (NGF) and local free radical insult [30,34,35]. Many of these elements can be implicated in damage to the vulnerable small fibers of peripheral nerves. Genome-wide association studies have also provided potential biomarkers. Genetic factors are possibly responsible for up to 50% of the disease susceptibility. Genes that were found to be potentially associated with fibromyalgia are *SLC64A4*, *TRPV2*, *MYT1L*, and *NRXN3*. Epigenetic alterations have also come into consideration as a triggering factor. In particular, FM appears to be characterized by a hypomethylated DNA pattern, in genes implicated in stress response, DNA repair, autonomic system response, and subcortical neuronal abnormalities [36,37]. In spite of the abundance of research dedicated to elucidating the molecular mechanisms responsible for triggering and maintaining as well as exacerbating FM, many questions still remain unanswered, and analgesic and therapeutic options remain limited. The neural pathogenesis of FM is complex and poorly understood as well. The current consensus is, however, that many precipitating factors contribute to the overall presentation of FM.

Current evidence suggests that FM belongs to a much larger continuum of chronic pain syndromes, which includes chronic fatigue syndrome, irritable bowel syndrome and other functional gastrointestinal syndromes, temporomandibular syndrome, migraine, and interstitial cystitis/bladder pain syndrome, among others, all with considerable overlap. In fact, the proposed new description for ICD-11 places it under the parent code for “Chronic widespread pain.” Estimates suggest that at least 2% of the adult population in the United States may be affected by FM, with an overall annual impact considering work absenteeism, lost productivity, and health care rivaling the costs of rheumatoid arthritis [38,39,40]. It has been noted that there is a great deal of overlap in symptoms between FM and SFN and an increasing number of studies have found the presence of SFN in patients carrying a diagnosis of FM as summarized in Table 6.

### Pain in Fibromyalgia

Pain is defined as an unpleasant sensory and emotional experience associated with, or resembling that associated with, actual or potential tissue damage. Chronic pain is defined as pain that lasts or recurs for more than three months [51,52]. It is an expression of neuronal plasticity involving complex interactions of many different peripheral and central structures from the skin surface to the cerebral cortex. Nociception is the mechanism whereby noxious stimuli are transmitted to the CNS. Broadly speaking, pain can be divided into 2 categories: adaptive and maladaptive. Adaptive pain is protective and contributes to survival by mitigating injury or promoting healing after injury has occurred. Maladaptive pain, in contrast, is an expression of the pathologic operation of the nervous system; it is pain as dysfunction for which there is no benefit to the organism [53].

Pain is further characterized as nociceptive, inflammatory, neuropathic, and functional. Nociceptive pain is in response to a noxious stimulus and is typically transient. Acute nociceptive pain (posttraumatic, postoperative, associated with acute disease) has an adaptive character. Inflammatory pain results in spontaneous pain and hypersensitivity to pain in response to tissue damage and inflammation. Neuropathic pain also results in spontaneous pain and hypersensitivity to painful stimuli, though is due to damage or a lesion of the nervous system (i.e., nerve ligation, axotomy). While both neuropathic and inflammatory pain may manifest as allodynia and hyperalgesia, inflammatory pain hypersensitivity returns to normal if the disease process is controlled. Alternatively, neuropathic pain persists long after the initiating event has healed and is an expression of pathological operation of the nervous system rather than a reaction to pathology [11]. Functional pain is hypersensitivity to pain resulting from abnormal central processing in the setting of normal input and tissues [53]. Pathological pain (of different mechanisms-neuropathic, inflammatory, functional) is maladaptive and may be chronic. These pain types are not exclusive of each other and will often be experienced simultaneously. For example, migraine is an episodic neurologic condition related to abnormal cortical activity that alters sensory input from dural and cerebrovascular sensory fibers and is associated with an abnormal sensory processing in the brain stem [53,54,55,56,57]. It possesses features of inflammatory and functional pain, as well as of objective neurologic dysfunction. Multiple mechanisms contribute to pain of all types, each of which is subject to or an expression of neural plasticity; the capacity of neurons to change their function, chemical profile, or structure [11]. Moreover, neurochemical correlates of the different types of pain also vary. For instance, in inflammation both Substance P (SP) and Calcitonin Gene Related Peptide (CGRP) are increased in the spinal cord, while the opposite is true in nerve ligation and axotomy [34,35,58,59,60,61].

Fibromyalgia (FM) is likely largely neuropathic based pain. Affected individuals elicit both hyperalgesia and allodynia. It is thought although not established that transduction in FM is primarily via two main subtypes of sensory axons; that being A delta fibers which typically yield sharp initial pain and polymodal C-fibers which transmit “second pain” of a dull aching or burning quality and persists longer than the noxious stimulus.

## 5. Discussion

Approximately 2%−5% of the adult population in the United States are affected by FM, with a female to male ratio of 2.3:1 according to the ACR 2010 modified guidelines [62]. Many correlates have been identified between Small fiber neuropathy (SFN) and Fibromyalgia (FM). In prior studies as many as 30–76% of patients carrying a diagnosis of FM have been found to have SFN using data collected from intraepidermal nerve fiber density (IENFD) values determined by skin punch biopsies. A recent systematic review and meta-analysis demonstrated the pooled prevalence of SFN in FM to be 49% [27]. This prevalence provides compelling evidence of a distinct phenotype involving SFN in FM, however, this phenotype has yet to be fully defined [47,63]. Recently it has been observed that the extent of small fiber pathology may in fact be related to symptom severity in FMS [42]. A recent study evaluating the estimated prevalence of self-reported neuropathic pain and small-fiber pathology in FM patients that met criteria for FM as well as those that had typical FM symptoms but did not fulfill criteria found that 62.4% of those that met criteria and 21% of those that did not, indicated that they had severe neuropathic pain. Of these individuals only 43.6% had neurophysiological investigations performed and only 1.9% received skin punch biopsies as well as only 13.2% received anti-neuropathic pain medications [64]. These findings highlight the degree to which SFN goes undiagnosed in these individuals and the effect that it has on their management.

Fibromyalgia is a clinical syndrome characterized by a dysregulation of neuroendocrine function and/or nociceptive processing, these characteristics have also been associated with SFN, so it comes as no surprise that there is evidence of SFN in people with FM. The hallmark features of FM include widespread pain and tenderness in reproducible anatomic locations, and many suffer sleep disturbances as well [65,66,67,68]. Autonomic dysfunction is also observed including irritable bowel, and orthostatic intolerance which typically presents as dizziness, lightheadedness, palpitations, muscle weakness, fatigue and headache without orthostatic hypotension [69]. Many of these symptoms can be potentially explained by small fiber pathology. Though there are many working theories regarding the pathophysiology of these symptoms, there has yet to be a biomarker that can easily and reliably be measured in these patients. FM remains undiagnosed in as many as 3 out of 4 people with the condition, with an average of 5 years between the time of onset of symptoms to diagnosis [70]. Many feel shame and hopelessness as a result of not having any objective measure of their condition. These conditions lead to increased medical expenses and disability and an overall lack of satisfaction in both patients and providers [39,71,72]. Indeed, quantifying SFN in patients with an FM diagnosis can provide guidance for further diagnostic studies and can facilitate better interventions and pharmacotherapy.

It has been noted in patients that are found to have SFN from other causes that their symptoms may pre-date a positive skin biopsy. This begs the question of whether this may be the case with SFN in FM as well. The recent observation of severity being related to confirmation of SFN supports this idea that as the symptoms worsen so too may the damage that is being done to the nerves. Perhaps there is some pathological process in the tissues that results in irritation and damage of the small nerve fibers that when left unabated eventually leads to the destruction of these vulnerable axons. These questions are important to consider in future research efforts and IENFD can provide a reliable end point measure for future studies in this area.

Therapy for FM is made difficult by the lack of consistent, evidence-based management guidelines. Pain processing requires transmission from peripheral tissues to the brain and can be modulated by both endogenous and exogenous processes. A multimodal approach with physical, psychological and pharmacologic interventions can help to modulate the perception of pain and improve function in affected individuals. Current treatment protocols involve physical therapy aimed at improved conditioning as well as pain management. Agents that modulate the neurochemical pain pathways present in the brain are preferred and thus various combinations of tricyclic antidepressants, selective serotonin reuptake inhibitors (SSRI), serotonin norepinephrine reuptake inhibitors (SNRI) and alpha 2 delta ligands are used. Unfortunately, treatment often fails to lead to adequate recovery, frustrating both the patient and physician [59,65,66,67,68]. Opioids are to be avoided and with long term use can contribute to central sensitization. Evaluation of SFN will allow for an objective measurement of the effects of interventions and the information provided by the skin biopsies will further research on the inflammatory and immunological processes involved. Additionally, studies have shown improvement with specific rehabilitative treatments in SFN and this body of work may prove equally applicable to those with FM [73,74,75].

Evaluating SFN can assist in further classification of FM as well. It has been suggested that some patients in whom FM is diagnosed and are found to have SFN, may in fact have another cause for their SFN. Identifying SFN in patients with FM then allows for additional diagnostic studies to determine whether or not there is an alternative cause for the SFN and in doing so guides management. It has also been suggested that FM is perhaps a general term for a spectrum of diseases that have yet to be fully identified and defined. It is entirely possible that there is a centrally mediated process at cause in some cases of FM and a peripherally mediated process at cause in others and perhaps even at times a combination of the two. Additionally, some investigators have described that complex regional pain syndrome/reflex sympathetic dystrophy (CRPS-I/RSD) may actually be a form of SFN [2].

Whatever the case may be, evaluating for SFN allows clinicians to further classify the individual’s disease process and thus provide better care.

## 6. Conclusions

The NIH defines biomarkers as: “characteristics that can be objectively measured and evaluated as an indication of normal or pathogenic processes or pharmacological responses to a therapeutic intervention [76].” Taking this definition into consideration, it is clear that nerve fiber density as evaluated through skin biopsy is the current gold standard for evaluation of SFN in those clinically presenting with symptoms of the disease and it may be used in combination with other tests to increase its diagnostic yield. Additionally, the evaluation of SFN in FM patients is a tool for identifying and further classifying a condition with no known biomarkers at present. As a result of this rapidly evolving body of research, it is our opinion that SFN should be considered in all patients with wide-spread body pain and thus should be considered in patients with Fibromyalgia.

## Figures and Tables

**Table 1:** Causes of Small Fiber Neuropathy.

Causes of Small Fiber Neuropathy
Diabetes and impaired glucose tolerance
Rapid glycemic lowering
Hyperlipidemia, metabolic syndrome
Chronic Kidney Disease
HIV, Hep C
Celiac disease, gluten sensitivity, inflammatory bowel disease (IBD)
Hypothyroidism, autoimmune thyroiditis
Vitamin B12 deficiency, Vitamin B1 deficiency, Vitamin B 6 toxicity
Paraproteinemia (MGUS)
Amyloidosis-familial amyloid polyneuropathy/TTR mutation, primary AL
Systemic lupus erythematosus, Sjogren syndrome, sarcoidosis, vasculitis, rheumatoid arthritis, Churg-Strauss Disease
Other immune mediated TS-HDS, FGFR-3, Plexin D1, Anti-voltage gated potassium channel (VGKC) antibody
Paraneoplastic syndromes (CRMP-5, PCA-2)
Hereditary-Fabry disease, SCN9A/10A/11A mutations, HSAN, Ehlers-Danlos syndrome
Pompe disease, Tangier disease
Toxic—Alcoholism, chemotherapy, thallium, metronidazole, nitrofurantoin, linezolid, statins, trauma (electrical, cold)
Pain syndromes—sickle cell disease, CRPS Type 1 (RSD)
Idiopathic

**Table 2:** Clinical procedure for skin punch biopsy for use in evaluation of IENFD.

Skin Punch Biopsy Procedure for Evaluation of Small Fiber Neuropathy	
*Typically, two biopsy sites are chosen and in total the procedure should take 15–30 min.*	
	1. Clean biopsy area with alcohol pad or equivalent antiseptic (e.g., chlorhexidine)
	2. Inject a sub-cutaneous 1–2 cm bevel of lidocaine/epinephrine in an apex proximal pattern around biopsy site being mindful to not inject directly over biopsy site.
	3. 3 mm punch is then inserted into the biopsy site, rotating as you push done and allowing the blade to cut the tissue. a. Keep tool perpendicular to the skin b. insert to the level of the cutis.
	4. Remove tissue gently with forceps and place each sample in small vial of Zamboni fixative (4% paraformaldehyde and picric acid) which can be kept at room temperature.
	5. Clean biopsy site and place gauze and tape over the biopsy site, keep dry for 24 h. Stitches are not necessary. Dressing is then removed within 12–24 h and full healing occurs after 7–10 days. In most cases the biopsy site is undetectable after a few months.

**Table 3:** Comparison of QSART, QST and IENFD in the evaluation of SFN.

	QSART	QST	IENFD
**Sensitivity**	80%	60–85%	88–95%
**Specificity**	Unknown	81%	89–97%
**Utility**	Sensitive and reproducible test for autonomic dysfunction	Available, well tolerated	Available anywhere, can measure proximal to distal gradient
**Availability**	Restricted to autonomic labs	Restricted—time consuming	Widespread: Can order commercial kit to any location
**Limitations**	Affected by anticholinergics and other drugs, requires time and special equipment	Variable and requires patient cooperation.	Invasive
**Disease surveillance?**	Yes	Unclear	Yes

**Table 4:** Widespread pain index and Symptom severity scale scoring breakdown. Together they make up the fibromyalgia symptoms scale (FS).

Widespread Pain Index (WPI) Score: Note the Number of Areas in Which the Patient Has Had Pain Over the Last Week. In How Many Areas Has the Patient Had Pain? (0–19 Points)		
*Left upper region (Region 1)*	*Right upper region (Region 2)*	*Axial region (Region 5)*
Jaw, left	Jaw, right	Neck
Shoulder girdle, left	Shoulder girdle, right	Upper back
Upper arm, left	Upper arm, right	Lower back
Lower arm, left	Lower arm, right	Chest
		Abdomen
*Left lower region (Region 3)*	*Right lower region (Region 4)*	
Hip (buttock, trochanter), left	Hip (buttock, trochanter), right	
Upper leg, left	Upper leg, right	
Lower leg, left	Lower leg, right	
**Symptom severity scale (SSS) score**		
1. Fatigue		
2. Waking unrefreshed		
3. Cognitive symptoms		
For each of the 3 symptoms above, indicate the level of severity over the past week using the following scale:		
0 = No problem		
1 = Slight or mild problems, generally mild or intermittent		
2 = Moderate, considerable problems, often present and/or at moderate level		
3 = Severe, pervasive, continuous, life-disturbing problems		
**The symptom severity score:** is the sum of the severity scores of the above mentioned symptoms (0–9) plus the sum of the number of the following symptoms the patient has been bothered by that occurred during the previous 6 months (0–3):		
1. Headaches (0–1)		
2. Pain or cramps in lower abdomen (0–1)		
3. Depression (0–1)		
The final symptom severity score is between 0–12		

**The fibromyalgia severity scale (FS)** is the sum of the WPI and SSS (also known as the polysymptomatic distress (PSD) scale).

**Table 5:** Summary of ACR Fibromyalgia diagnostic criteria revisions.

The Evolution of the ACR Diagnostic Criteria for Fibromyalgia
**ACR 1990 Criteria**
*Widespread pain in combination with:*
◆ Tenderness at 11 or more of 18 specific tender points
◆ Digital palpation should be done with about 4 kg of force
◆ The patient must state that the palpation was painful for the tender point to be considered positive
◆ Patient must not have any other disorder that might otherwise explain the pain
**ACR 2010 Criteria**
*Tender point examination is eliminated and replaced with patient questionnaire that involves two scales: The wide spread pain index (WPI) and the symptom severity score (SSS)—the diagnosis was made when:*
◆ WPI 7 or greater and SSS 5 or greater -or-
◆ WPI 3–6 and SSS 9 or greater
**ACR 2011 Criteria Modification**
*Physician-estimate of somatic symptoms is eliminated and the WPI and SSS are revised and combined to create the 0–31 FM symptoms scale (FS) which includes:*
◆ 19 pain locations
◆ 6 self-reported symptoms, including difficulty sleeping, fatigue, poor cognition, headache, depression and abdominal pain
◆ An FS score of 13 or greater best classified patient that either met or did not meet the 2010 criteria
**ACR 2016 Criteria (current)**
*A diagnosis of Fibromyalgia is valid irrespective of other diagnoses. It does not exclude the presence of other clinically important illnesses. A diagnosis is made when all three of the following criteria are met:*
◆ WPI 7 or greater and SSS 5 or greater -or- WPI 4–6 and SSS 9 or greater
◆ Generalized pain, defined as pain in at least 4 of 5 regions, is present
◆ Symptoms have been present at a similar level for at least 3 months

**Table 6:** Summary of studies that have demonstrated the presence of SFN in patients diagnosed with FM [41,42,43,44,45,46,47,48,49,50].

Author	Year	Country	Sample Size	Study Group	Group Size	Mean Age	Sex (Female/Male)	Diagnostic Technique	Guideline	Prevalence #	Prevalence %
DeTommaso et al.	2014	Italy	81	FibromyalgiaControl	21 60	51 +/− 9 53 +/− 6	18/3 50/10	Skin biopsy: thigh and distal leg	2010 ACR	16	76
Evdokimov et al.	2019	Germany	248	FibromyalgiaMD w/ P Control	117 11 120	52 (22–75) 52 (43–58) 50 (20–84)	117 * 11 * 120 *	Cornel confocal microscopy/Skin biopsy: thigh and distal leg	2010 ACR	76	63
Giannoccaro et al.	2013	Italy	52	FibromyalgiaControl	20 32	40 +/− 6 Unavailable	19/1 Unavailable	Skin biopsy: thigh and distal leg	1990 ACR	6	30
Kosmidis et al.	2014	Greece	80	FibromyalgiaControl	46 34	53 (29–76) 32 (19–84)	41/5	Skin biopsy: distal leg	2010 ACR	16	34
Lawson et al.	2018	USA	155	Fibromyalgia	155	49 +/− 12	105/50	Skin biopsy: thigh and distal leg	2010 ACR	62	40
Leinders et al.	2016	Germany	116	FibromyalgiaControl	28 88	51 (39–74) 44 (16–79)	26/2 80/8	Skin biopsy: thigh or distal leg	1990 ACR	14	50
Oaklander et al.	2013	USA	57	FibromyalgiaControl	27 30	47 (26–68) 45 (25–65)	20/7 24/6	Skin biopsy: distal leg	2010 ACR	11	41
Oudejans et al.	2016	Netherlands	-	FibromyalgiaControl	39	39 (19–58) Unavailable	36/3 Unavailable	Corneal confocal microscopy	1990 or 2010 ACR	20	51
Ramirez et al.	2015	Mexico	34	FibromyalgiaControl	17 17	44 +/− 5 43 +/− 6	17 * 17 *	Corneal confocal microscopy	1990 or 2010 ACR	12	71
Uceyler et al.	2013	Germany	155	FibromyalgiaMD w/o P Control	24 10 121	59 (50–70) (39–75) Unavailable	22/2 9/1 Unavailable	Skin biopsy: thigh and distal leg	1990 ACR	10	42

* Subjects were exclusively female. MD w/P: Major depression pain, MD w/o P: Major depression without pain.
